# Risk Factors for Apathy in Polish Patients with Parkinson’s Disease

**DOI:** 10.3390/ijerph181910196

**Published:** 2021-09-28

**Authors:** Agnieszka Gorzkowska, Joanna Cholewa, Jaroslaw Cholewa, Aleksander Wilk, Aleksandra Klimkowicz-Mrowiec

**Affiliations:** 1 Department of Neurorehabilitation, Faculty of Medical Sciences in Katowice, Medical University of Silesia, 40-055 Katowice, Poland; agorzkowska@sum.edu.pl; 2 Department of Physical Education and Adapted Physical Activity, The Jerzy Kukuczka Academy of Physical Education in Katowice, 40-065 Katowice, Poland; a.cholewa@awf.katowice.pl; 3 Department of Health Related Physical Activity and Tourism, The Jerzy Kukuczka Academy of Physical Education in Katowice, 40-065 Katowice, Poland; 4 Department of Neurosurgery, University Hospital, 31-501 Krakow, Poland; wialeksander@gmail.com; 5 Department of Internal Medicine and Gerontology, Faculty of Medicine, Medical College, Jagiellonian University, 31-008 Krakow, Poland; Aleksandra.Klimkowicz@mp.pl

**Keywords:** Parkinson’s disease, apathy, risk factors, brain-derived neurotrophic factor

## Abstract

Apathy, a feeling of indifference or a general lack of interest and motivation to engage in activity, is one of the most common neuropsychiatric symptoms in Parkinson’s disease (PD). The large variation in prevalence and the underlying pathophysiological processes remain unclear due to heterogeneous PD populations. The purpose of this study was to identify risk factors for apathy, the modification or treatment of which may be clinically relevant and improve quality of life and caregiver burden for patients with Parkinson’s disease. Caucasian subjects with Parkinson’s disease were included in the study. Baseline demographics, neurological deficit, medications taken, cognitive and neuropsychiatric status, and the polymorphisms in the brain-derived neurotrophic factor gene were assessed. Apathy was diagnosed in 53 (50.5%) patients. They were less educated (OR 0.76 CI 0.64–0.89; *p* = 0.001), more frequently depressed (OR 1.08 CI 1.01–1.15; *p* = 0.018), and less frequently treated with inhibitors of monoamine oxidase-B (MAOB-I) (OR 0.07 CI 0.01–0.69; *p* = 0.023). Although apathetic patients were more likely to carry the Met/Met genotype, differences in the brain-derived neurotrophic factor *BDNF* rs6265 polymorphism between apathetic and non-apathetic PD patients were not statistically significant in multivariate analysis. Some risk factors for apathy may be clinically modifiable. Further studies are needed to assess whether modeling modifiable apathy risk factors will affect the prevalence of this neuropsychiatric symptom in patients with Parkinson’s disease.

## 1. Introduction

Parkinson’s disease (PD) is characterized by motor symptoms, such as bradykinesia, tremors, and rigidity. However, it may be preceded and often accompanied by a variety of neuropsychiatric and cognitive symptoms. These are of great clinical importance, affecting quality of life and increasing the need for hospitalization and treatment.

Apathy, or a feeling of indifference or a general lack of interest in the outside world and motivation to be active, is one of the most bothersome psychiatric symptoms in PD [1]. It refers to a complex of behavioral, cognitive, and emotional disturbances [2].

Apathy occurs in 16.5–62.3% of patients with PD and is primarily associated with older age, a lower Mini Mental State Examination score, depression, a higher Unified Parkinson’s Disease Rating Scale motor score, and greater disability [3].

Data from pharmacological [4] and genetic studies [5,6] suggest that the pathophysiology of apathy may be explained by dysfunction of the dopaminergic system. BDNF (brain-derived neurotrophic factor), a major member of the neurotrophin family, is widely expressed in the mammalian brain; maintains the survival of dopaminergic neurons; and promotes synaptic plasticity, dendrite morphogenesis, arborization, and neurogenesis in adult brains. The highest levels of BDNF are found in the hippocampus followed by the cortex, regions that are involved in many neuropsychiatric diseases [7]. Neuroimaging studies have shown an association between the Val66Met polymorphism of *BDNF* gene and gray matter volume in the anterior cingulate cortex and dorsolateral prefrontal cortex [8], brain regions that have been linked to the pathogenesis of apathy [9]. However, the association between *BDNF* gene polymorphisms and apathy has never been studied before.

The aim of the current study was to identify risk factors for apathy. Modifying, preventing, or treating such factors may have clinical relevance and improve the quality of life of patients with PD.

## 2. Materials and Methods

### 2.1. Study Population

Silesian Medical University patients with PD were invited to participate in the study. The duration of recruitment to the study was six months. All patients meeting the study criteria were recruited. Participants had to meet diagnostic criteria for idiopathic PD. Exclusion criteria were as follows: dopaminergic replacement therapy (DRT) treatment < 6 months prior to study inclusion; clinical diagnosis of dementia (Mini-Mental State Examination (MMSE) <25 points, Clock Drawing Test (CDT) <10 points); comorbid movement disorders; secondary causes of PD; and neurosurgical treatment (deep brain stimulation or pallidotomy). PD was diagnosed using the United Kingdom Parkinson’s Disease Society Brain Bank criteria [10]. Demographic information was collected, including age; education; gender; disease duration; the use of dopaminergic medications; and comorbidities e.g., hypertension, diabetes, cardiovascular disease (ischemic heart disease and cardiac arrhythmias). Motor symptoms were assessed using Part III of the Movement Disorder Society Unified Parkinson’s Disease Rating Scale (MDS-UPDRS) [11], and a modified Hoehn–Yahr scale [12] was assigned.

All participants were Caucasian and of Eastern European descent. The work was conducted in accordance with the Declaration of Helsinki. The medical ethical committee of the Academy of Physical Education in Katowice approved the study (approval code:6/2013). Informed written consent was provided by each patient and was included in the study.

### 2.2. Neuropsychiatric Assessment

Patients completed cognitive screening using the MMSE [13] and the CDT [14] for global cognitive measure. Apathy was assessed with the Sterkstein Apathy Scale (AS) [15], and mood was assessed with the Beck Depression Inventory (BDI) [16].

AS is a validated 14-item questionnaire specifically designed to assess apathy in PD. The AS contains questions about daily life over the past four weeks related to various manifestations of apathy. Each question is read aloud by the researcher, and the patient has a choice of four possible answers: “not at all”; “a little”; “some”; or “a lot”. Each answer is scored from 0 to 3 points. The total score ranges from 0 to 42 points, with a cut-off value of ≥14 points indicating a higher likelihood of apathy (sensitivity 66% and specificity 100%) [15].

The BDI assesses symptoms of depression experienced during the past week. It is a self-monitoring questionnaire consisting of 21 multiple-choice questions. Each answer is rated on a scale of 0 to 3. The patient can score from 0–63 points. A cut-off value of >14 points is recommended to diagnose depression in PD [16]. The BDI is considered to have excellent reliability and accuracy for use in patients with PD and is recommended by the American Academy of Neurology (AAN) and the Quality Standards Subcommittee of AAN as a screening tool for depression in this patient population [17].

### 2.3. Genotyping

Deoxyribonucleic acid (DNA) was extracted from peripheral blood samples from 105 PD patients using a GeneMATRIX Quick Blood DNA Purification Kit (EURx Sp. z o.o., Gdansk, Poland) and subsequently standardized to uniform concentration (20 ng/μL) using a NanoDrop ND-1000 spectrophotometer (Thermo Scientific, Wilmington, DE, USA). All samples were genotyped for the presence of common missense single-nucleotide polymorphisms (SNPs) within the *BDNF* gene (rs6265, c.196G > A, Val66Met) using a pre-validated allelic discrimination TaqMan real-time polymerase chain reaction (PCR) assay (Assay ID: C__11592758_10) and TaqMan GTXpress Master Mix (Life Technologies, Carlsbad, CA, USA). Fluorescence data were captured using an ABI PRISM 7500 FAST Real-Time PCR System (Applied Biosystems, Foster City, CA, USA) after 40 cycles of PCR [18].

### 2.4. Statistical Analyses

All statistical analyses were performed using STATISTICA for Windows version 12 (StatSoft Inc., Tulsa, OK, USA). First, associations were found between apathy and predisposing factors (including genotype and allele frequencies of *BDNF* SNPs). Odds ratios (OR) with 95% confidence intervals (CI) and their *p* values were obtained using univariate logistic regression. Variables significantly associated in the univariate logistic regression were then entered into the multivariate logistic regression analysis. The final predictive model for apathy was fitted using the forward stepwise selection method. The significance level was set at *p* < 0.05.

## 3. Results

A total of 105 consecutive PD patients (45 females and 60 males, mean age 65.31 ± 8.98) were included in the study. Apathy was diagnosed in 53 patients (50.48%). Univariate analysis showed significant correlations between apathy, lower education, and cardiovascular disease. UPDRS part III scale scores were significantly higher among apathetic patients. Apathetic patients were significantly less frequently treated with MAOB-I. The clinical characteristics of the study group are shown in Table 1.

Patients with and without apathy differed significantly in the distribution of the rs6265 polymorphism. Patients with apathy more often carried the A allele than non-apathetic patients. Table 2 presents the results.

Multivariable logistic regression analysis based on the results of univariate logistic regression was performed. The best predictive model identified the following predictors for apathy: a shorter education time (OR 0.76 (0.64–0.89) and *p* < 0.001); higher BDI score (OR 1.08 (1.01–1.15), *p* = 0.018); and less frequent treatment with MAOB-I (OR 0.07 (0.01–0.69), *p* = 0.023). The A allele of the *BDNF* gene was no longer significant.

## 4. Discussion

Our study confirmed some of the previously obtained results. We found, similar to the work of Cubo et al. [19], that lower education is a risk factor for apathy in PD patients. Apathy was described early in the course of the disease, preceding motor symptoms. Thus, it can be hypothesized that lower education may be a consequence of apathy rather than a cause of it, exhibited long before the clinical manifestation of symptoms as the occurrence of less activity, including activity directed at acquiring education.

Consistent with previous research [3,20], it was observed that depression was an independent risk factor for apathy. Apathy often coexists with depression but can occur independently of depression in PD [21,22]. Although the clinical manifestations of both disorders are similar, they are distinct syndromes in which mood is the main differentiating parameter—neutral in apathy and negative in depression. Therefore, it is important to screen for both apathy and depression in order to categorize patients into the appropriate group.

New in this study is the observation that MAOB-I treatment significantly reduces the risk of apathy in PD patients. These findings are supported by the study of Kirsch-Darrow et al. [21], who showed that when compared with PD patients receiving levodopa or dopamine agonists in monotherapy, MAOB-I as an adjunct to levodopa showed a more favorable effect on apathy, depression, and quality of life.

It was found in the current study that a higher score on the UPDRS motor scale was associated with apathy in the univariate factor analysis. Patients with apathy had a higher score of just over 6 points than patients without apathy. However, in a multivariate analysis, this difference proved to be insignificant. In comparison, in a meta-analysis including 374 patients from eight cohorts, patients with apathy had a 6.5-point higher UPDRS motor scale score than patients without apathy, and this difference was statistically significant [3].

Whether the *BDNF* gene Val66Met polymorphism is a genetic risk factor for many neuropsychiatric diseases has been studied in different ethnic groups, and the results are inconclusive [23]. The association between *BDNF* gene polymorphisms and apathy has not been studied before. The *BDNF* gene Val66Met (G to A) SNPs results in the conversion of valine to methionine at position 66 in the pro-domain region. This polymorphism reduces the distribution of BDNF in dendrites, limits its transport to secretory granules, and impairs the activity-dependent secretory pathway of BDNF [24,25]. Although positive associations between the *BDNF* gene Val66Met polymorphism and cognitive and neuropsychiatric symptoms have been previously reported, no such associations were found in the present study. Patients carrying the AA (Met/Met) genotype of the *BDNF* polymorphism were more likely to be apathetic, but these results were not significant in logistic regression analysis.

In the current study, there were no differences between apathetic and non-apathetic patients in terms of age. The two groups also did not differ in terms of MMSE scale score. A meta-analysis of studies on risk factors for apathy in patients with PD [3] found that patients with apathy are on average 3.3 years older than patients without apathy. Our groups differed in age by only 1.5 years. Commenting on the lack of differences in the MMSE, it may be recalled that since apathy co-occurs with dementia, patients with dementia were excluded from the study to avoid bias, and groups by design should not differ in this respect.

## 5. Limitations

A limitation of our study was the relatively small number of subjects included. It cannot be ignored that with a larger number of subjects, statistical differences between groups would prove statistically significant. Moreover, we analyzed only one SNPs variant of the *BDNF* gene as a potential genetic risk factor.

## 6. Conclusions

Apathy remains one of the most common and disabling non-motor symptoms in PD. Its pathophysiology remains largely unclear but appears to be multifactorial. Our study confirmed the role of lower education and depression in the occurrence of apathy in PD patients, and for the first time demonstrated an association between MAOB-I treatment and a reduced risk of apathy in PD patients.

The study also showed a potential association of apathy in PD with *BDNF* gene Val66Met polymorphisms, which needs to be confirmed in further studies.

## Figures and Tables

**Table 1:** Demographic and clinical characteristics of the participants group.

Criteria	Apathetic PD Patients *n* = 54	Non-Apathetic PD Patients *n* = 51	*p* Value
Age (SD)	66.07 (9.06)	64.51 (8.87)	0.373
Women (%)	20 (37)	25 (49)	0.219
Education (years) (SD)	11.96 (2.95)	13.98 (2.50)	0.001
BMI ≥30 *n* (%)	13 (24.07	7 (13.73)	0.185
BMI (SD)	26.88 (4.02)	26.37 (3.40)	0.489
Hypertension *n* (%)	26 (48.15)	24 (47.06)	0.911
Diabetes *n* (%)	6 (11.11)	10 (19.61)	0.234
Cardiovascular disease *n* (%)	19 (35.19)	9 (17.65)	0.048
UPDRS III (points)	28.11 (14.05)	21.90 (11.44)	0.02
Hoehn–Yahr scale (points)	2.33 (0.64)	2.24 (0.60)	0.422
Dyskinesia *n* (%)	10 (18.52)	13 (25.49)	0.392
Fluctuation ON/OFF *n* (%)	23 (42.59)	21 (41.18)	0.884
L-dopa start therapy *n* (%)	43 (79.63)	39 (76.47)	0.697
DA start therapy *n* (%)	7 (12.96)	5 (9.80)	0.613
MAOB-I start therapy *n* (%)	3 (5.56)	5 (9.80)	0.42
L-dopa current therapy *n* (%)	51 (94.44)	51 (96.08)	0.697
Dopamine agonists current therapy *n* (%)	27 (50.00)	30 (58.82)	0.367
MAOB-I current therapy *n* (%)	1 (1.85)	10 (19.61)	0.019
LED (mg)	784.43 (450.46)	769.61 (423.29)	0.926
Treatment duration (y)	5.66 (3.75)	5.96 (4.38)	0.71
Time to start treatment (y)	1.11 (1.41)	1.53 (2.20)	0.255
MMSE (points)	27.35 (2.08)	28.10 (1.94)	0.065
BDI (points)	12.74 (8.14)	8.57 (6.89)	0.009

BMI—body mass index; UPDRS—Unified Parkinson’s Disease Rating Scale; MAOB-I—inhibitors of monoamine oxidase-B (selegiline 5 mg once daily); LED—levodopa equivalent dose; MMSE—Mini Mental State Examination; BDI—Beck Depression Inventory; *p* value—univariate logistic regression.

**Table 2:** The *BDNF* rs6265 G > A genotype in apathetic and non-apathetic PD patients.

	Apathetic PD Patients	Non-Apathetic PD Patients	All Groups	*p* Value
allele				
A (%)	82 (77.36)	93 (89.42)	175 (83.33)	0.0178
G (%)	24 (22.64)	11 (10.58)	35 (16.66)	
genotype				
AA (%)	6 (11.11)	1 (1.96)	7 (6.67)	
GA (%)	13 (24.07)	8 (15.69)	21 (20.0)	0.03
GG (%)	35 (64.81)	42 (82.35)	77 (73.33)	

*p* value—univariate logistic regression.

## Data Availability

The data presented in this study are available on request from the corresponding author. The data are not publicly available due to privacy.

## References

[1] Barone P., Antonini A., Colosimo C., Marconi R., Morgante L., Avarello T.P., Bottacchi E., Cannas A., Ceravolo G., Ceravolo G. (2009). The PRIAMO study: A multicenter assessment of nonmotor symptoms and their impact on quality of life in Parkinson’s disease. Mov. Disord..

[2] Robert P., Onyike C.U., Leentjens A.F., Dujardin K., Aalten P., Starkstein S., Verhey F.R., Yessavage J., Clement J.P., Drapier D. (2009). Proposed diagnostic criteria for apathy in Alzheimer’s disease and other neuropsychiatric disorders. Eur. Psychiatry.

[3] den Brok M.G., van Dalen J.W., van Gool W.A., van Charante E.P.M., de Bie R.M., Richard E. (2015). Apathy in Parkinson’s disease: A systematic review and meta-analysis. Mov. Disord..

[4] Zhang L., Zhou F.M., Dani J.A. (2004). Cholinergic drugs for Alzheimer’s disease enhance in vitro dopamine release. Mol. Pharmacol..

[5] Mitaki S., Isomura M., Maniwa K., Yamasaki M., Nagai A., Nabika T., Yamaguchi S. (2013). Apathy is associated with a single-nucleotide polymorphism in a dopamine-related gene. Neurosci. Lett..

[6] Somme J.H., Molano Salazar A., Gonzalez A., Tijero B., Berganzo K., Lezcano E., Fernandez Martinez M., Zarranz J.J., Gómez-Esteban J.C. (2015). Cognitive and behavioral symptoms in Parkinson’s disease patients with the G2019S and R1441G mutations of the LRRK2 gene. Parkinsonism Relat. Disord..

[7] Hofer M., Pagliusi S.R., Hohn A., Leibrock J., Barde Y.A. (1990). Regional distribution of brain-derived neurotrophic factor mRNA in the adult mouse brain. EMBO J..

[8] Matsuo K., Walss-Bass C., Nery F.G., Nicoletti M.A., Hatch J.P., Frey B.N., Monkul E.S., Zunta-Soares G.B., Bowden C.L., Escamilla M.A. (2009). Neuronal correlates of brain-derived neurotrophic factor Val66Met polymorphism and morphometric abnormalities in bipolar disorder. Neuropsychopharmacology.

[9] Santangelo G., D’Iorio A., Maggi G., Cuoco S., Pellecchia M.T., Amboni M., Barone P., Vitale C. (2018). Cognitive correlates of “pure apathy” in Parkinson’s disease. Parkinsonism Relat. Disord..

[10] Gibb W.R., Lees A.J. (1988). The relevance of the Lewy body to the pathogenesis of idiopathic Parkinson’s disease. J. Neurol. Neurosurg. Psychiatry.

[11] Goetz C.G., Tilley B.C., Shaftman S.R., Stebbins G.T., Fahn S., Martinez-Martin P., Poewe W., Sampaio C., Stern M.B., Dodel R. (2008). Movement Disorder Society-sponsored revision of the Unified Parkinson’s Disease Rating Scale (MDS-UPDRS): Scale presentation and clinimetric testing results. Mov. Disord..

[12] Hoehn M.M., Yahr M.D. (1967). Parkinsonism: Onset, progression and mortality. Neurology.

[13] Folstein M.F., Folstein S.E., McHugh R.R. (1975). ‘Mini Mental State’: A practical method for grading the cognitive state of patients for the clinician. J. Psychiatr. Res..

[14] Sunderland T., Hill J.L., Mellow A.M., Lawlor B.A., Gundersheimer J., Newhouse P.A., Grafman J.H. (1989). Clock drawing in Alzheimer’s disease. A novel measure of dementia severity. J. Am. Geriatr. Soc..

[15] Starkstein S.E., Mayberg H.S., Preziosi T.J., Andrezejewski P., Leiguarda R., Robinson R.G. (1992). Reliability, validity, and clinical correlates of apathy in Parkinson’s disease. J. Neuropsychiatry Clin. Neurosci..

[16] Schrag A., Barone P., Brown R.G. (2007). Depression rating scales in Parkinson’s disease: Critique and recommendations. Mov. Disord..

[17] Miyasaki J.M., Shannon K., Voon V., Ravina B., Kleiner-Fisman G., Anderson K., Shulman L.M., Gronseth G., Weiner W.J. (2006). Practice Parameter: Evaluation and treatment of depression, psychosis, and dementia in Parkinson disease (an evidence-based review): Report of the Quality Standards Subcommittee of the American Academy of Neurology. Neurology.

[18] Hook B., Schagat T. Isolating Genomic DNA from Large Volumes of Buffy Coat using Maxwell^®^ 16. Promega Corporation Web. Site. http://pl.promega.com/resources/pubhub/isolation-of-genomic-cdna-from-large-volume-of-buffy-coat-using-maxwell-16/.

[19] Cubo E., Benito-León J., Coronell C., Armesto D., ANIMO Study Group (2012). Clinical correlates of apathy in patients recently diagnosed with Parkinson’s disease: The ANIMO study. Neuroepidemiology.

[20] Brown D.S., Barrett M.J., Flanigan J.L., Sperling S.A. (2019). Clinical and demographic correlates of apathy in Parkinson’s disease. J. Neurol..

[21] Kirsch-Darrow L., Marsiske M., Okun M.S., Bauer R., Bowers D. (2011). Apathy and depression: Separate factors in Parkinson’s disease. J. Int. Neuropsychol. Soc..

[22] Gorzkowska A., Cholewa J., Małecki A., Klimkowicz-Mrowiec A., Cholewa J. (2020). What Determines Spontaneous Physical Activity in Patients with Parkinson’s Disease?. J. Clin. Med..

[23] Tsai S.J. (2018). Critical Issues in BDNF Val66Met Genetic Studies of Neuropsychiatric Disorders. Front. Mol. Neurosci..

[24] Chen Z.Y., Jing D., Bath K.G., Ieraci A., Khan T., Siao C.J., Herrera D.G., Toth M., Yang C., McEwen B.S. (2006). Genetic variant BDNF (Val66Met) polymorphism alters anxiety-related behavior. Science.

[25] Baquet Z.C., Bickford P.C., Jones K.R. (2005). Brain-derived neurotrophic factor is required for the establishment of the proper number of dopaminergic neurons in the substantia nigra pars compacta. J. Neurosci..

